# Disease course and treatment effects of a JAK inhibitor in a patient with CANDLE syndrome

**DOI:** 10.1186/s12969-019-0322-9

**Published:** 2019-05-02

**Authors:** M. Boyadzhiev, L. Marinov, V. Boyadzhiev, V. Iotova, I. Aksentijevich, S. Hambleton

**Affiliations:** 1 Department of Pediatrics, Medical University, Varna, Bulgaria; 2 National Human Genome Research Institute, National Institutes of Health, Washington D.C, USA; 3 Instituste of Cellular Medicine, Newcastle University, Newcastle Upon Tyne, UK

**Keywords:** Autoinflammation, CANDLE syndrome, Immunoproteasome, JAK inhibitors, Nephrolithiasis

## Abstract

**Background:**

CANDLE syndrome (an acronym for Chronic Atypical Neutrophilic Dermatosis with Lipodystrophy and Elevated Temperature) is a recently described rare autosomal recessive disorder charaterized by systemic autoinflammation. Clinical manifestations include presentation in the first year of life, episodes of fever accompanied by erythematous skin lesions, progressive lipodystrophy, violaceous periorbital swelling and failure to thrive. This syndrome is caused by loss of function mutations and malfunction of the immunoproteasome complex. Most patients have biallelic mutations in the PSMB8 gene that encodes the β5i catalytic subunit of the immunoproteasome. Examples of digenic inheritance have been also described in CANDLE. CANDLE patients have strong type I interferon gene expression signature and they are responsive to treatment with JAK inhibitors. However, possible serious side-effects remain a concern. Here, we report another patient with CANDLE whose disease activity was well controlled by the treatment with baricitinib.

**Case presentation:**

We report a Bulgarian patient of the Turkish ancestry who carries biallelic mutations in the PSMB8 gene: p.Ala92Val and p.Lys105Gln. The pathogenic variant p.Ala92Val has not been previously described in patients with CANDLE. We also comment on the unusual feature in this patient, nephrolithiasis, that has not been described in other patients, however it might be related to the positive family history for kidney stones. We have treated the patient with the JAK inhibitor baricitinib for the past year and we observed a significant amelioration of his inflammatory episodes, skin and joint manifestations, and improvements in physical activities and growth. The treatment with glucocorticoids (GC) was completely discontinued. No side effects have been observed, however they remain in consideration for a life-long therapy of this disease.

**Conclusions:**

CANDLE should be suspected in patients with early-onset systemic inflammatory disease and prominent skin manifestations. Molecular testing can confirm the clinical diagnosis and is very important in guiding therapies. Treatment with JAK inhibitors is highly efficacious and appears to be safe in children with CANDLE and other intereforonopathies.

## Background

Chronic atypical neutrophilic dermatosis with lipodystrophy and elevated temperature (CANDLE) syndrome has been described by Torello et al. in 2010 [1]. CANDLE syndrome patients present in early-infancy, often during the neonatal period, with symptoms of recurrent fevers and a prominent skin rash [1, 2]. The rash tends to be exacerbated during inflammatory episodes. Torello et al. described 4 children between 2 and 14 years of age, presenting with annular purpuric plaques mainly on the body. The authors made a thorough investigation of the skin lesions through repeated skin biopsies [3]. The periorbital swelling also appears during the first months of life. Growth retardation and failure to thrive are some of the most striking features of the syndrome.

The loss of fat tissue (lipodystrophy) mostly in the upper limbs and face becomes visible later in the disease progress. The lack of fat tissue over the cheeks gives patients a typical “aging” look. The abdominal fat tissue, on the contrary, tends to be increased, which leads to metabolic disturbances [2].

Arthralgia and arthritis are almost always present although with variable degrees [4]. Myalgia, myositis and muscle wasting also can lead to reduced patients‘mobility. Other not so common features are clubbed fingers, hepatomegaly and splenomegaly, hypertrichosis, acanthosis, alopecia areata, arterial hypertension, basal ganglia calcification [5]. Roberts et al. reported a South African girl with microdontia and microstomia [6]. The laboratory findings in patients with CANDLE include increased inflammatory markers, CRP, ESR, and marked leukocytosis [7]. Hypertrigliceridemia is a common finding.

The first disease-causing mutations were identified in the PSMB8 gene and were associated with functional dysregulation of the immunoproteasome complex. Subsequently, mutations in additional genes encoding both immunoproteasome and constitutive proteasome subunits were identified: PSMB4, PSMA3, PSMB9. More recently, heterozygous de novo mutations in the regulatory POMP protein have been reported in patients with autoimmunity, immunodeficiency and autoinflammation resembling CANDLE. Thus the inheritance pattern in CANDLE can be autosomal recessive or in the form of a de novo mutation.

Two more syndromes with a striking resemblence to the CANDLE syndrome have been reported - Nakajo-Nishimura syndrome (NNS) was first reported in 1939 by Dr. Nakajo [8]. In 1950, Nishimura reported three more cases with similar features. In 2010, Garg et al. [9] reported three adult patients with a disease they named Joint Contractures, Muscle atrophy, microcytic anemia, and Panniculitis-induced lipodystrophy syndrome (JMP). Patients diagnosed with NNS and JMP carry biallelic either homozygous or compound heterozygous mutations in PSMB8. Currently there is no consensus classification of these three syndromes. Some authors use the term PRAAS (proteasome-associated autoinflammatory syndrome), others - ALDD (autoinflammation, lipodystrophy, and dermatitis) [10]. The exact prevalence of CANDLE syndrome is yet unknown.

PRAAS is caused by a dysregulation in the proteasome-immunoproteasome function [11, 12] Immunoproteasomes are found mainly in immune cells. Their structure is very similar to that of the proteasomes [13–15]. Immunoproteasome formation is mainly induced by proinflammatory cytokines such as IFNs and in response to increased demand for protein degradation in cells [16, 17].

The production of IFNs is increased during viral infections or other types of stress. This leads to increased protein degradation by the immunoproteasomes. When they are not working properly, the pathogen-derived proteins and other damaged proteins accumulate in cells stimulating the production of IFNs. That forms a vicious circle leading to persistent inflammatory state. Since JAK-STAT signalling pathway is activating type 1 and 2 IFNs, it is not surprising that wider and more frequent use of JAK inhibitors in clinical studies leads to remarkable results [18–20]. As mentioned above, in the case of CANDLE syndrome, there is a defect in the immunoproteasome function that leads to activation of the IFNs through the JAK-STAT signaling pathway. The expected efficacy of JAK inhibitors is due to the action they exert on that exact pathway. The long-term prognosis remains unclear because of the recent description and the very limited treatment experience.

## Case presentation

We present a 6 years old boy, presented soon after birth with erythematous eruption and hemorrhagic blisters (pernio), initially on the palms. Subsequently the rash spread over the entire body, accompanied with solitary subcutaneous nodules and violaceous periorbital swelling. His rash was worse during fever episodes. He was hospitalized multiple times at a tertiary pediatric unit with the clinical picture of severe inflammation. No immunisations were administered after the age of 3 months [21]. Three consecutive skin biopsies were performed from active efflorescences. They showed inflammation with the presence of lymphocytes, neutrophils, macrophages and mastocytes (CD117+). Some parts showed leukocytoclasis. Several autoimmune and autoinflammatory syndromes were considered in the differential diagnosis, including Sweet syndrome, Mevalonate kinase deficiency, CINCA/NOMID and leukocytoclastic vasculitis.

At the age of 1 year 5 months, he was started on methylprednisolone 1 mg/kg.d-1 with a reasonable effect on the rash and fevers. However, all attempts to taper the dose led to immediate relapse of the symptoms. Succesively, hydroxycloroquine and methotrexate were attempted as GC sparing agents, but they were soon withdrawn due to lack of any clinical effect.

**Fig. 1:**
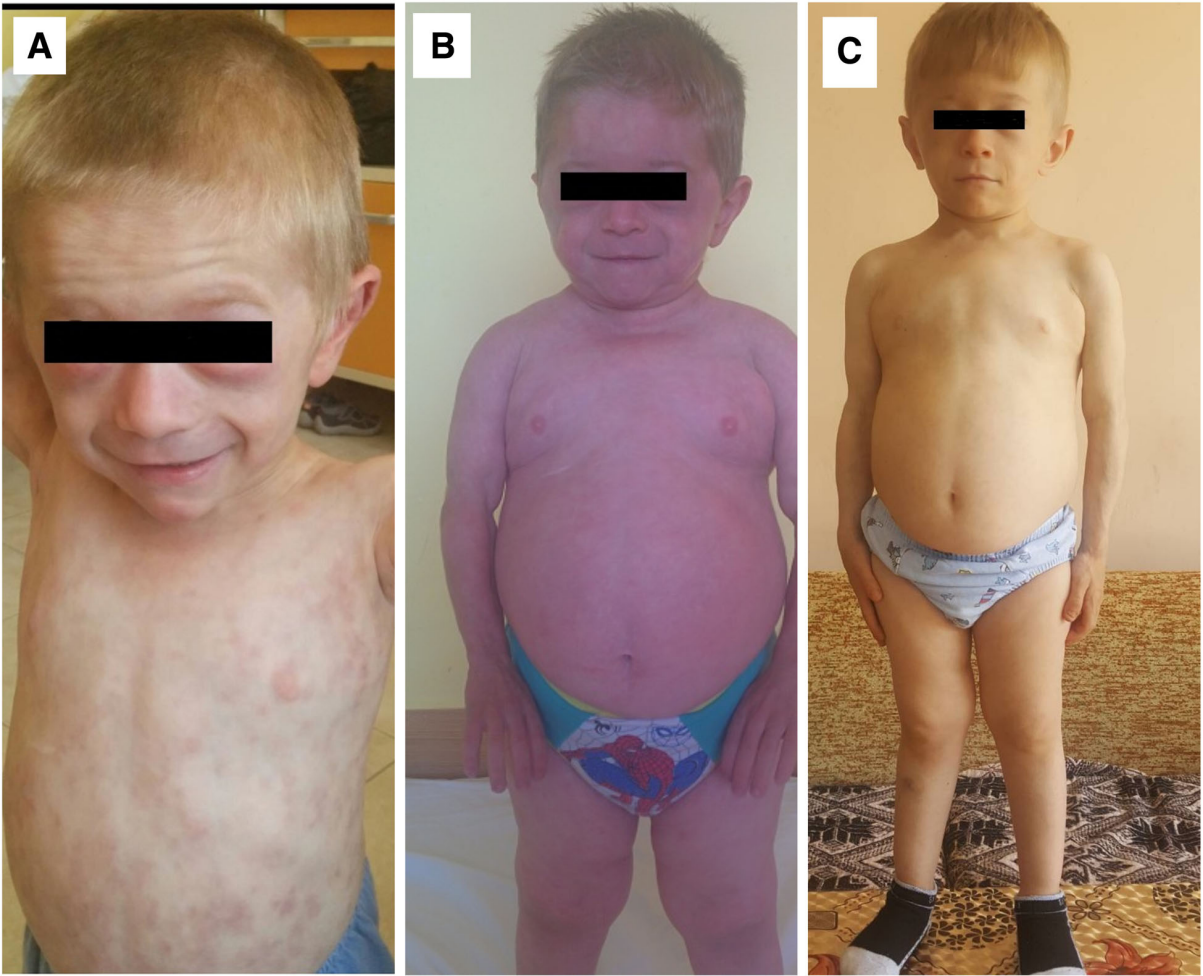
The patient: **a**. When he is 2 years 10 months old; **b**. When he is 5 years old, just before the start of baricitinib; **c**. After 7 months of treatment with baricitinib

The boy presented to us at the age of 2 years and 10 months. He was looking pretty unwell, with elevated daily temperatures reaching 39.9 °C, persistent skin eruption with nodules, livid and red papules and macules on the head, body, upper and lower extremities, palms and soles. Purple periorbital swelling was also present (Fig. 1). Hypertrichosis was noted all over the body. Subcutaneous adipose tissue was significant for lipodystrophy. The arms, shoulders and face had well demarcated outline of the muscles, which gave the false impression of increased muscularity. The parents recalled episodes of joint pain and swelling of knees and fingers, but there was no objective evidence of contractures or joint space narrowing. The face was characteristic for atrophic facial musculature and absence of buccal fat pads, thus giving him an appearance of an old-man. He also had hypertelorism and epicanthus. Other symptoms were cough, tachydyspnea and hepatomegaly. The patient appeared very stunted for his age group. Age- and sex-specific growth evaluation showed significant short stature (− 4.1 SDS) and a decreased weight (− 2.3 SDS), www.cdc.gov/growthcharts. His bone age, according to Greulich and Pyle’s atlas, was 1 year 3 months (<− 2.0 SDS). Blood workup showed elevated CRP - 114.09 mg/L (0–5.0), neutrophilia, anemia with serum iron level < 2 mcmol/L (7.2–21.5). Thorough investigation showed no cardiac, pulmonary or neurologic involvment at the time.

CANDLE syndrome was suggested as the most probable diagnosis after discussion and evaluation of the patient’s medical history and current status. Blood samples from the child and both parents were sent to NIH for genetic testing. The patient was found to have compound heterozygous mutations in the PSMB8 gene (p.A92V/p.K105Q). Both parents are carriers of one mutation.

## Follow-up

After confirming the diagnosis of CANDLE, we intended to find a way to start therapy with JAK inhibitors. At the age of 3 years 6 months the boy presented with a violent flank pain. Ultrasound examination showed nephrolithiasis, with several small concrements in both kidneys. His mother reported a history of nephrolithiasis. Two months later, an abdominal ultrasound revealed mild hydronephrosis, without any sign of nephrolithiasis. This finding remains until the present time.

Other complications have appeared with time, related to the syndrome and the therapies. At 5 years of age despite the high doses of glucocorticoids, inflammatory flares with fever, and elevated CRP continued to be frequent – at least one episode every month. Failure to thrive was progressing. Arterial hypertension with the blood pressure reaching 160/100 mmHg and frequent headaches necessitated addition of ACE-inhibitor and calcium inhibitor to his therapy. Cholesterol was high for age (5.97 mmol/l).

Gastric complaints became frequent with stomach pain and vomiting at least once weekly. While on high doses of methylprednisolone his appetite became voracious. Despite the large food intake his height remained static, while growth velocity was less than 0.5 cm per year (Fig. 1). The patient complained of muscle pain, difficulty walking and easy fatigue.

The JAK 1/2 inhibitor baricitinib (Olumiant) was started shortly after the patient‘s 5th birthday. The NIH protocol was followed with a starting dose of 6 mg/day for the first 3 days, and after no side effects were observed the dose was increased to 8 mg/day. After 7 months of treatment we witnessed dramatic and objective improvements:

- No new episodes of systemic inflammation for the past 3 months: no fevers, malaise, and vomiting.
- The first attempt of stopping glucocorticoid treatment completely was undertaken 3 months after the start of baricitinib. The GC dose was quickly tapered down from 1 mg/kg/day to less than 0.1 mg/kg/day during the first month. This led to the new appearance of three violaceous subcutaneous nodules, resembling the typical rash for CANDLE on his foot and fingers), which disappeared in a week. Methylprednisolone treatment was completely stopped at 5th month after the initiation of treatment with baricitinib and without further relapses.
- Drastic improvement of the skin rash, and dissapearence of the periorbital swelling (Fig. 1)
- Normalisation of the blood pressure, allowing the withdrawal of the calcium inhibitor during the second month of treatment and the ACE-inhibitor after 7 months.
- Increased growth velocity - +8.8 cm/7 months, extrapolated growth velocity of 15 cm/year.
- Decreased abdominal circumference by 8 cm - from 65 cm to 57 cm.
- Improved mobility with no complaints of fatigue and/or muscle pain. Soon after the start of baricitinib treatment the patient was able to perform longer and more difficult physical activities.
- During 7 months of treatment the patient needed a 5 day-treatment with an antibiotic due to a common upper airways infection.

Shortly after the start of baricitinib the boy refused to walk, and complained of muscle pain in the thighs. No clinical or laboratory signs of myositis or arthritis were found. Complaints continued for a week, without worsening or further complications.

**Fig. 2:**
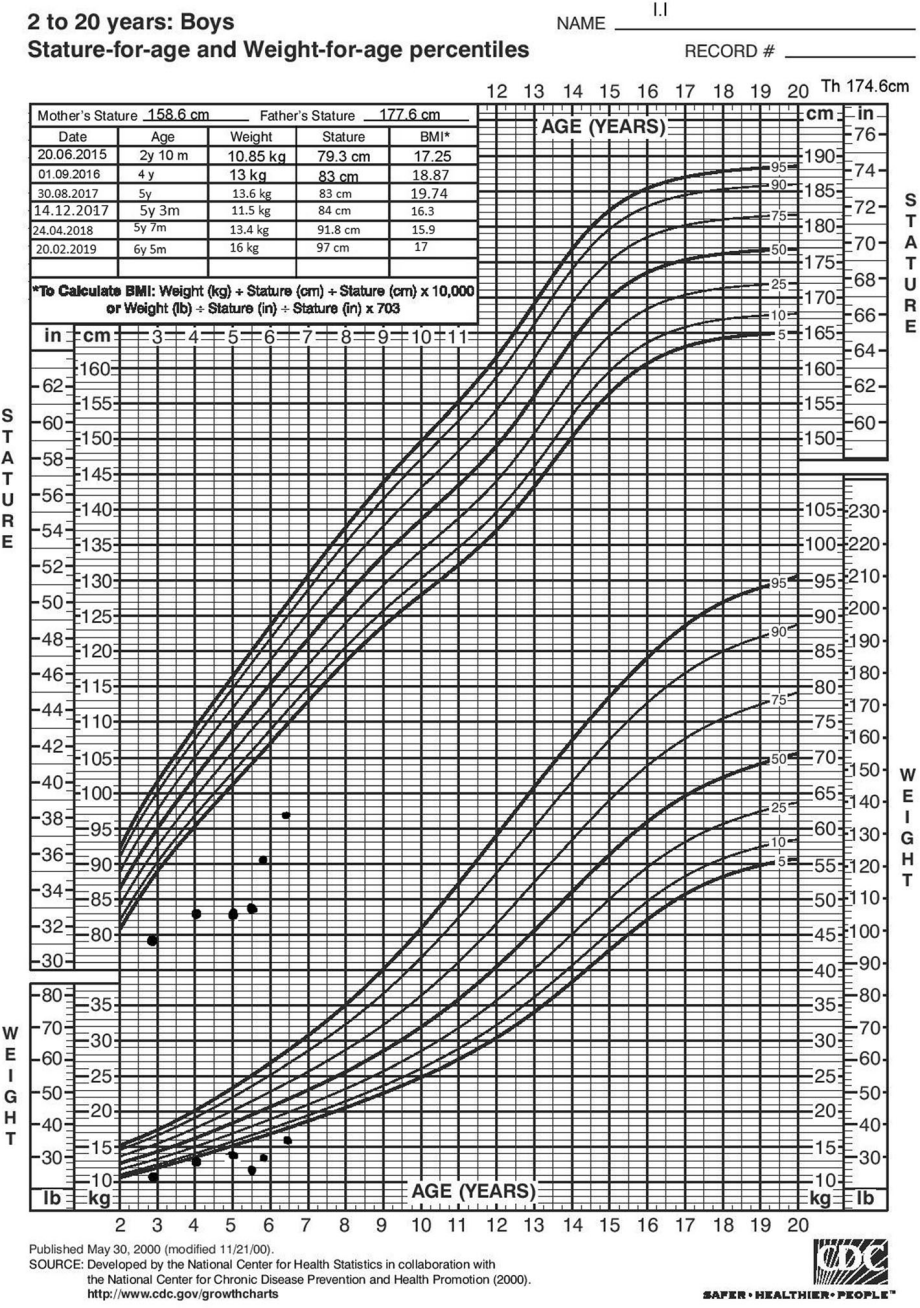
Growth chart of the patient

During the patient‘s visit on the seventh month after the start of baricitinib treatment he was feeling much better, with no pain while walking in contrast to his previous visits, with normalized appetite and increased physical endurance. Control blood count showed normal leucocytes and hemoglobin, and normal neutrophil count. Despite the lack of clinical symptoms, elevated CRP values were noted on two occasions, raising the question of the best dose of baricitinib (Table 1). The significant increase in his growth velocity has been documented (Fig. 2). Those findings repeated during the visit at the end of the first year after the start of baricitinib treatment (Fig. 2).

**Table 1:** Inflammation markers during the patient follow up

Date	Age	Leucocytes ×10^9^/L	Neutrophils ×10^9^/L	CRP mg/L
June 2015	2 y 10 mo	8.27	7.51	114.09
April 2016	3 y 8 mo	16.62	11.01	23.71
November 2016	4 y 3 mo	16.12	12.16	16.3
January 2017	4 y 5 mo	10.81	9.11	76.18
March 2017	4 y 7 mo	10.89	5.12	43.04
Start of Baricitinib in August 2017				
September 2017	5 y 0 mo	16.88	12.24	2.8
October 2017	5 y 2 mo	8.73	3.5	0.13
December 2017	5 y 4 mo	7.66	3.53	8.78
February 2018	5 y 5 mo	7.89	4.47	17.13
April 2018	5 y 7 mo	6.74	2.78	7.48
February 2019	6 y 5 mo	7.5	5.79	18.63

## Discussion

We present a young patient with molecularly confirmed CANDLE syndrome. The patient is compound heterozygous: he has two mutations in the PSMB8 gene (p.A92V/p.K105Q). One of the mutations – p.Ala92Val (c.275C > T) is novel and has not been reported, although another mutation affecting the same amino acid residue is reported in patients with CANDLE [22]. The second mutation p.Lys105Gln (c.313A > C; p.K105Q) was recently described in a report from Brehm et al. in 2015 [16].

The patient was treated with JAK 1/2 inhibitor, baricitinib, for a period of 1 year and 6 months without any side effects and with impressive improvement in all aspects of the disease.

JAK inhibitors are part of a new class of small molecule drugs and they have been used in the treatment of rare oncologic diseases and many autoimmune and autoinflammatory diseases. Janus Kinase inhibition has been used with a different degree of success in myeloproliferative diseases, lupus, psoriatic arthritis, inflammatory bowel diseases [23, 24]. They are especially efficient in the treatment of adult rheumatoid arthritis, showing promising results in comparison with biological agents. JAK inhibitors are also very convenient for the patients because of their oral intake.

Treatment effect of baricitinib described in this article is comparable to the previous published reports in CANDLE patients [18]. The mean dose of baricitinib in this study was 8.5 mg/day. Our patient is on a maintenance dose of 8 mg/day. However, increased values of CRP in our patient were detected during the last couple of visits. No other signs of infections were present. This indicates the presence of persisting subclinical inflammation and raises the question about the best dose of baricitinib.

Montealegre et al. described a decrease in the mean glucocorticoid dose by 73%, and 4 out of 12 patients managed to discontinue glucocorticoids completely. After 5 months of treatment in our patient, the GC treatment was discontinued completely and with no disease relapses.

Five out of six patients with myositis, described by the above referenced study, showed improvement in muscle fatigue and improved physical endurance as it has been the case with our patient [18]. Joint involvement, which is a typical clinical finding for most of the published CANDLE patients, was not a predominating feature in our case.

The study by Montealegre et al. also described 17 serious adverse events in 4 patients. Most common were those of infectious origin – infections of the upper respiratory tract, one probable Pneumocystis jiroveci pneumonia, Rotavirus and *Clostridium difficile* infections. One patient has been removed from the study due to a lack of efficacy and development of avascular necrosis. Our patient did not suffer from any serious complications connected to an infectious cause or any other adverse events.

Our patient showed persistently normal hemoglobin levels during the treatment, while two patients reported by Montealegre et al. developed anemia.

The results from the JAK inhibitor treatment of here described patient with CANDLE syndrome are consistent with the previously published cohort study, with catch-up growth and physical activity improving significantly more in our patient. The short-term effects of JAK 1/2 inhibitor according to our expirience are excellent with no side effects reported, though the long-term effectiveness and possible serious side-effects remain a concern. The increased values of CRP, suggesting persistent subclinical inflammation, raise the question if the best dose of baricitinib is 8 mg/day or higher. Tofacitinib has been used by other authors with beneficial effect [25] but we do not have experience with the drug.

Although glucocorticoids control the acute inflammation, the significant side effects make their long term use undesirable. We hope that JAK inhibitors will secure a long-term control of the disease symptoms without severe side-effects and a better quality of life of the children affected by CANDLE syndrome.

## Conclusion

The results from the JAK inhibitor treatment of here described patient with CANDLE syndrome are consistent with the previously published cohort study. Although the short-term effects of JAK 1/2 inhibitor are excellent, the long-term effectiveness and possible serious sideeffects remain a concern.

## Abbreviations

ALDD: Autoinflammation, lipodystrophy, and dermatitis
ANA: Antinuclear antibodies
CANDLE: Chronic atypical neutrophilic dermatosis with lipodystrophy and elevated temperature
CINCA: Chronic infantile neurological cutaneous and articular syndrome
CRP: C reactive protein
ESR: Erythrocyte sedimentation rate
GC: Glucocorticoids
IFN: Interferon
JAK: Janus kinase
JMP: Joint contractures, muscle atrophy, microcytic anemia, and panniculitis-induced lipodystrophy syndrome
NIH: National Institute of Health
NNS: Nakajo-Nishimura syndrome
NOMID: Neonatal onset multisistem inflammatory disease
PRAAS: Proteasome-associated autoinflammatory syndrome
